# Study protocol for a multi-level cross-sectional study on the equitable reach and implementation of coordinated specialty care for early psychosis

**DOI:** 10.1186/s43058-023-00476-6

**Published:** 2023-08-08

**Authors:** Oladunni Oluwoye, Ari Lissau, Sheldon Stokes, Alexandria T. Selloni, Najé James, Solmaz Amiri, Michael G. McDonell, Deidre M. Anglin

**Affiliations:** 1 Department of Community and Behavioral Health, Elson S. Floyd College of Medicine, Washington State University, 412 E. Spokane Falls Blvd, Spokane, WA 99210-1495 USA; 2 Department of Psychology, City College of New York, City University of New York, New York, USA; 3 Department of Medical Education and Clinical Sciences, Elson S. Floyd College of Medicine, Washington State University, Spokane, WA USA; 4 Institute for Research and Education to Advance Community Health, Washington State University, 1100 Olive Way, Ste 1200, Seattle, WA 98101 USA

**Keywords:** Contextual factors, Coordinated specialty care, Disparities, Dissemination, Mental health services, Reach

## Abstract

**Background:**

Approximately 115,000 young adults will experience their first episode of psychosis (FEP) each year in the USA. Coordinated specialty care (CSC) for early psychosis is an evidence-based early intervention model that has demonstrated effectiveness by improving quality of life and reducing psychiatric symptoms for many individuals. Over the last decade, there has significant increase in the implementation of CSC programs throughout the USA. However, prior research has revealed difficulties among individuals and their family members accessing CSC. Research has also shown that CSC programs often report the limited reach of their program to underserved populations and communities (e.g., ethnoracial minorities, rural and low socioeconomic neighborhoods). Dissemination and implementation research focused on the equitable reach and implementation of CSC is needed to address disparities at the individual level.

**Methods:**

The proposed study will create a novel integrative multi-level geospatial database of CSC programs implemented throughout the USA that will include program-level data (e.g., geocoded location, capacity, setting, role availability), provider-level data (race, ethnicity, professional credentials), and neighborhood-level census data (e.g., residential segregation, ethnic density, area deprivation, rural-urban continua, public transit time). This database will be used to characterize variations in CSC programs by geographical location and examine the overall reach CSC programs to specific communities. The quantitative data will be combined with qualitative data from state administrators, providers, and service users that will inform the development of dissemination tools, such as an interactive dashboard, that can aid decision making.

**Discussion:**

Findings from this study will highlight the impact of outer contextual determinants on implementation and reach of mental health services, and will serve to inform the future implementation of CSC programs with a primary focus on equity.

Contributions to the literature

- This study will create a novel integrative multi-level database of more than 346 coordinated specialty care programs for early psychosis that links provider-, program-, and neighborhood-level characteristics.
- This is the first study that uses a health equity lens to examine outer contextual factors and the potential reach of CSC programs.
- Findings from this study will inform future implementation of coordinated specialty care programs with specific attention to how policies and practices inform equitable access.

## Introduction

Nearly 10 million Americans live with a psychotic disorder, and of those, up to 115,000 individuals experience their first episode of psychosis (FEP) before age 25, each year in the USA [1–3]. Neighborhoods where individuals reside are important determinants of health. Neighborhood-level determinants cover a broad range of social, economic, and political conditions, including urbanicity; ethnic density (racial composition of communities); and residential stability, which are considered outer contextual factors that impact access to resources, opportunities, and services. The concept of *reach* in implementation science is often defined as the proportion of individuals who are willing to participate in services or who receive timely and adequate services [4–6]. Shelton et al. defined *reach* as the one component in RE-AIM that considers whether services equitably reach populations or communities and how neighborhood determinants may or may not influence reach [7]. The overall *reach* of mental health services to individuals in the early stages of psychosis may vary based on the neighborhood characteristics where individuals reside by shaping where services are implemented and the type of mental health services that are offered [8]. Prior research has demonstrated that historically minoritized and/or underserved communities have less access to mental health and specialty care services, resulting in disparities [9–11]. Outer contextual factors maintain systemic inequity in neighborhood-wide access to mental health care, including early intervention services for psychosis [12–15].

### Implementation of coordinated specialty care for early psychosis

Based on early intervention programs implemented in other countries (e.g., Great Britain, Canada, Australia), coordinated specialty care (CSC) models are designed to improve outcomes for FEP by providing multiple evidence-based interventions. The combination of evidence-based interventions is delivered by a multidisciplinary team and includes the following components: (1) family psychoeducation, (2) individual or group psychotherapy, (3) case management, (4) low doses of select antipsychotic medication, and (5) supported education and employment [16, 17]. The National Institute of Mental Health (NIMH) has stated that CSC models are the gold standard of care for early psychosis in the U.S.

To date, approximately 350 CSC programs have been implemented in 49 states across the U.S. [18, 19]. The increasing number of CSC program throughout the U.S. has been facilitated by the allocation of federal funds through the Community Mental Health Block Grant to support states in the scale-up of evidence-based early interventions such as CSC. Along with the increase in the number of CSC programs implemented is the growing body of evidence that has demonstrated the effectiveness of CSC on improving the quality of life and psychiatric symptoms throughout treatment [16, 17, 20, 21]. Over the years, CSC programs have been adapted, creating variations in size, additional services offered, and team roles, yet very little is known about the current organizational-level landscape of CSC programs. Given the expansion of CSC programs, it is also unclear where programs are most likely to be geographically located and whether certain communities have the potential to access spatially distributed opportunities and whether CSC equitably reach communities that have been underserved. Without this knowledge to guide future decision-making on implementation of CSC programs, inequities in access, service utilization, and treatment outcomes will continue to exist.

### Dissemination and implementation science to reduce inequities in mental health services

Dissemination and implementation (D&I) science provides an underutilized approach to address inequities and disparities in mental health care, particularly as it pertains to access and reach of services. Dissemination science seeks to develop and understand strategies that facilitate the spread of evidence-based interventions to stakeholders [22]. Work in this area has largely focused on the translation of evidence for policymakers to assist in the decision-making process and inform mental health policies [23–25]. Yet, to the authors’ knowledge, there has been very limited dissemination research that has primarily focused on addressing disparities by translating evidence to inform equitable decision-making.

Implementation science has been defined as the study of methods or strategies that promote the uptake of evidence-based practices into routine practice, with the primary goal of improving the quality and effectiveness of health services [26]. Components of implementation science have largely focused on whether services are delivered as intended and whether evidence-based interventions or models are staffed as intended to provide optimal care based on evidence of effectiveness. These intentions are often influenced by contextual factors which are a unique set of factors, circumstances, or characteristics of the environment that surround implementation efforts [27, 28]. Existing determinant frameworks such as the Theoretical Domains Framework [29, 30] and the Consolidated Framework For Implementation Research [31] refer to inner (organizational environment—e.g., culture, readiness) and outer (wider environment beyond organization—e.g., local infrastructure, physical environment) contextual factors as important constructs that may hinder or facilitate implementation. Research has demonstrated the relationship between inner contextual factors and implementation outcomes in a variety of settings [32–34], but less emphasis has been placed on understanding the relationship between outer contextual factors and implementation [27, 35]. Through an equity lens, purposeful research is needed that considers how outer contextual factors such as neighborhood-level determinants may impact how programs are staffed, the type of services offered, and how services are delivered, all of which are vital to the implementation and sustainability or programs.

## Methods

### Study aims

The current study utilizes concepts from dissemination and implementation science to understand and address potential inequities in the reach (accessibility and availability) of CSC throughout the U.S. The study aims are:

1. To characterize the variation in program-level characteristics of CSC by geographical location
2. To examine the reach of CSC programs, using geographical accessibility as an indicator, based neighborhood-level characteristics
3. To develop an interactive dashboard using web-mapping technology that will translate evidence to enable decision makers and community stakeholders to make evidence-informed decisions on program implementation to expand reach of services equitably

### Conceptual framework

The National Institute of Minority Health and Health Disparities (NIMHD) Research Framework organizes levels of determinants (individual, interpersonal, neighborhood, societal) by domains of influence (biological, behavioral, physical/built environment, sociocultural environment, health care system), using a socio-ecological approach [36]. It offers a systematic approach to identify and locate influences that may be especially relevant to understanding inequities in care [37]. Previous research has focused on individual- (e.g., demographics) and interpersonal-level determinants (e.g., family members) and the interaction with biological and behavioral domains to understand onset of psychosis-like experiences, CSC service utilization, and treatment outcomes [21, 38, 39].

Neighborhood-level determinants influence inequities in mental health services and outcomes. The physical/built environment encompasses the community environment. Rurality and low socioeconomic area deprivation are neighborhood-level determinants that have been associated with the limited distribution of mental health resources in these areas [10, 40]. The sociocultural environment, which includes local structural discrimination, racial residential segregation, and ethnic density, is often considered as proxies for racism. There are well-known associations between residential segregation and ethnic density, and mental health disparities, yet no study to date has examined residential segregation and ethnic density effects on the distribution of CSC programs. Limited geographic accessibility may contribute to these well-known disparities and may be a driving factor that impacts the reach of CSC. Within the healthcare system domain, there has been a scale up and expansion of CSC; however, very little is known about organizational resources such as treatment setting, role availability, and provider characteristics of CSC programs and the variability that may exist throughout the U.S. Taken together, the proposed project focuses on outer context of CSC through neighborhood-level determinants in the physical/built environment (e.g., rural/urban, area deprivation) and sociocultural environment (e.g., ethnic density, residential segregation), the inner context of CSC (e.g., location, treatment capacity, size, provider characteristics), and the relationship that these contextual factors have with the availability and accessibility of CSC programs.

### Study design

**Fig. 1:**
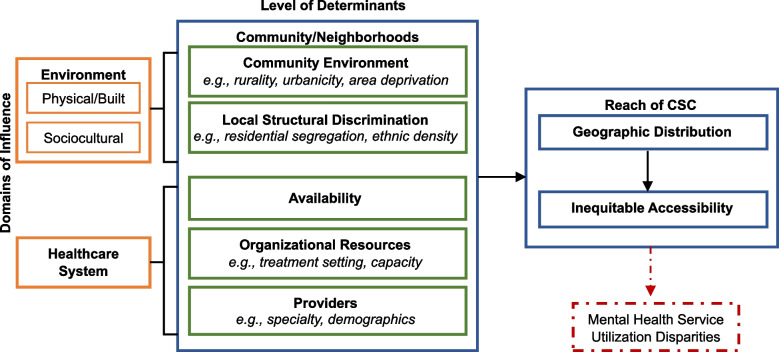
Conceptual framework

Guided by the conceptual framework, illustrated in Fig. 1, we will use a multi-level cross-sectional design that utilizes data from multiple sources, whereby facility-level data will be collected from > 346 CSC programs across the U.S., which will then be merged with neighborhood-level Census data based on the geographic location of CSC programs. In aim 1, we will characterize CSC programs based on facility-level data and understand the geographic distribution by neighborhood-level factors. In aim 2, we will examine the association between neighborhood-level factors and access to CSC programs to identify geographic disparities. In aim 3, we will conduct qualitative interviews with mental health administrators and policy makers, CSC providers, and service users and their family members/support persons. Quantitative and qualitative findings will be used to inform the development of an interactive dashboard with web mapping visualization capabilities to disseminate findings from aims 1 and 2. The goal of our interactive dashboard is to aid policy and decision makers on the placement of CSC programs, in an effort to provide equitable access.

### Quantitative survey and measures

To contextualize information about CSC programs, aggregated client- and provider-level data and program-level characteristics will be collected using a REDCap-based survey (Table 1). Program-level characteristics will be gathered using modified questions from the Mental Health Services Questionnaire [41]. The physical location of each program will be geocoded using ESRI ArcGIS Business Analyst USA Local Composite geocoder and R software. Programs will be asked to provide information on program size, such as client capacity, services offered, and number of team members. Programs will also be asked to provide information on the types of funding they receive (e.g., Community Mental Health Block Grants, donor funds) as well as what forms of payment or non-payment (e.g., Medicaid, private, free services) are accepted. Additional questions will be asked on provider-level characteristics, such as race and ethnicity, age, and professional background, and client-level aggregated sociodemographic information on clients currently served.

**Table 1:** Table of measures and indicators

**Domains and constructs**	**Level and constructs**	**Source**	**Measure, definition, categories**	**Aim**
	**Enabling facility-level factors**			
**Healthcare system**	Location	Directory	Number, street, direction, city, state, zip code	Aims 1, 2, and 3
	*CSC program-level characteristics*			
	Size	Survey	Capacity (number), service delivery team (number)	Aim 1
	Setting	Survey	Hospital, neighborhood outpatient, university-hospital clinic	Aim 1
	Funding Source	Survey	Block grant funds, hospital, university, donors, federal grants, other.	Aim 1
	Payment	Survey	Private, public, no insurance, self-payment	Aim 1
	Services	Survey	E.g., occupational therapy, telehealth services, translators	Aim 1
	Referrals	Survey	E.g., emergency room, family member, criminal justice system	Aim 1
	*Provider-level demographics*			
	Race/ethnicity	Survey	Non-Hispanic Black, non-Hispanic White, Asian, Pacific Islander, American Indian/Alaska Native, Latinx	Aim 1
	Professional credentials	Survey	E.g., licensed social worker, clinical psychologist, medical doctor, mental health counselor	Aim 1
	Gender	Survey	E.g., cis male, transgender female	Aim 1
	*Client-level characteristics*			
	Race/ethnicity	Survey	Non-Hispanic Black, non-Hispanic White, Asian, Pacific Islander, American Indian/Alaska Native, Latinx	Aim 1
	Gender	Survey	E.g., Cis Male, Transgender Female	Aim 1
	Age	Survey	Mean age	Aim 1
	Language	Survey	% of non-English speaking; % of monolingual Spanish	Aim 1
	**Contextual neighborhood-level factors**			
	Geographic accessibility	Survey 2019 ACS	Availability of CSC programs relative to the population size, proximity, time	Aims 2 and 3
**Physical/built environment**	Rurality/urbanization	RUCA	Metropolitan, micropolitan, rural	Aims 1, 2, and 3
	Socioeconomic area deprivation	2019 ACS	Neighborhood concentration disadvantage, concentrated poverty	Aims 1, 2, and 3
	Broadband internet	2019 ACS	Availability of broadband internet subscription rates	Aims 2 and 3
	Public transportation	2019 ACS	Public transit time	Aims 2 and 3
**Sociocultural environment**	Residential segregation	2019 ACS	Index of dissimilarity, separation index	Aims 1, 2, and 3
	Ethnic density	2019 ACS	Neighborhood-level racial/ethnic composition	Aims 1, 2, and 3

*RUCA* Rural-urban commuting areas, *ACS*, US Census Bureau American Neighborhood Survey, *CSC* Coordinated specialty care, *CMHB* Community Mental Health Block grant

### Survey recruitment and data collection procedures

Trained research staff will contact each CSC program listed in the integrated directory (by phone call or emails) to confirm and obtain the physical address of the CSC program and to answer survey questions. Programs will be provided with an option to complete the survey over the phone or through a REDCap-based survey link, which will be emailed to the point of contact person, preferably program directors. To address weaknesses in the rigor of prior research and to ensure a high response rate for programs, the following strategies will be used: (1) an initial email request will be sent to CSC programs from the investigative team; (2) phone calls/emails to known state-level administrators will be conducted to increase buy-in and participation from CSC programs in the state; (3) follow-up calls for clarification on the study and to collect additional/missing information with program directors will be performed by research staff; (4) respondents will be compensated with a $30 e-gift card for completing the online survey; (5) reminder calls/emails will be sent to respondents to complete the survey. It is anticipated that by the end of the data collection period, there will be a final response rate of 80%.

### Semi-structured interviews

Approximately 80 qualitative interviews will be conducted to contextualize quantitative findings and to inform the development of a national GIS, using web mapping and visualization technology. Interviews will be conducted with 25 state-level mental health administrators and policy makers involved in the implementation of CSC in their state, 25 CSC providers to gather insight on the potential barriers and facilitators to the reach of their CSC, and 30 interviews with service users of CSC and their family members to understand community assets and needs. A purposeful sampling strategy will be used to recruit participants to achieve a balance between increasing inference quality/trustworthiness (internal validity) and generalizability/transferability (external validity) [42]. Consent forms will be emailed and reviewed with each state and local policymaker or service user and family member using e-consent procedures. Interviews will be conducted virtually using an online meeting platform (e.g., Zoom) or by phone and will last approximately one hour. Participants will be remunerated $50.

## Data analysis

### Descriptive analysis for aim 1

To characterize CSC programs within each state and collectively, program-level data will be analyzed descriptively with means and standard deviations (*SDs*) for continuous measures (i.e., size), frequencies (*N*), and percentages for categorical measures (i.e., setting, funding source, payment, provider and client characteristics). Medians and interquartile ranges will be provided for non-symmetric distributions, as necessary. Data will also be stratified by setting type to identify potential differences using simple linear regression for each program-level characteristic. *T*-test will be used to determine whether an association exists (i.e., the regression coefficient is non-zero) between facility capacity, service delivery team size, the number and types of services offered, the average proportion of provider race/ethnicity and sex/gender, the average proportion of client race, client sex/gender, non-English speaking clients, average client age, and the rurality of neighborhood (census block groups). Chi-square tests will be performed to assess the association of setting, funding source, payment, referral type, and rurality. All analyses are unadjusted for potential confounding factors. Inferential results will be presented as unstandardized regression coefficients with 95% confidence intervals. The alpha error rate to 0.05 will be set as the threshold for statistical significance. All analyses will be conducted using STATA version 14.2 or the R statistical software when applicable.

### Multi-level and spatial analysis for aim 2

To examine reach, three measures will be used to calculate geographic accessibility. First, distance from block groups to the closest CSC program will be calculated. Second, transit time from urban block groups to the closest CSC program will be estimated. Third, a two-step floating catchment area method with a distance decay function will be used [43]. This technique accounts for the availability and capacity of CSC programs relative to the population size, proximity of CSC programs relative to block groups, and time as a potential barrier within catchment areas [44, 45]. The physical location of CSC programs will be geocoded (i.e., latitude and longitude). Travel time will be measured using the ESRI ArcGIS Network Analyst between the geographic coordinates of the street intersection points, representing the CSC program and block group population weighted centroids. Considering both speed limits and turn-by-turn calculations, the Network Analyst tool will estimate travel time. Block groups will be represented by the latitude and longitude of population weighted centroids that capture how populations are distributed in block groups. The two-step floating catchment area method uses floating, overlapping catchment areas. This method includes a distance decay function equivalent to recognizing that population groups are not equally likely to be accessing services from both nearby and up to the catchment boundaries which denotes that the interaction between population and facilities decreases as distance between them increase [43, 46].

Analyses will be conducted at the census block group level. Using the geocoded address for CSC programs from aim 1, the number of CSC programs per population (*N* (%)) will be summarized stratified on rural-urban continua, socioeconomic area deprivation, residential segregation, and ethnic density. To bridge the gap between aim 1 and aim 2, measures of central tendency and variability (i.e., means and standard deviations (*SDs*) for continuous measures (i.e., size), frequencies (*N*) and percentages for categorical measures) will be used to describe availability by rural-urban continua, socioeconomic area deprivation, residential segregation, and ethnic density of the census block groups in which the CSC programs are available. The median and IQR will be calculated in cases where variable distributions are found to be asymmetric. Neighborhood-level factors that likely impact the accessibility of CSC programs will be modeled. Key covariates including rural-urban continua, socioeconomic area deprivation, residential segregation, and ethnic density will be introduced into the model. Appropriate random effects will be used to account for the clustering of counties within states and neighborhoods within counties to yield valid variance estimates. Effects of rurality, neighborhood disadvantage, racial composition (e.g., percent of Hispanic and African American populations), areas with broadband internet shortages, and access to public transportation will be investigated. Multilevel modeling (i.e., mixed effects modeling or hierarchical modeling) will be conducted separately for each neighborhood-level characteristic (i.e., rural-urban continua, socioeconomic area deprivation, residential segregation, ethnic density). Following the unadjusted models, a multilevel model with all main effects for neighborhood-level factors will be fit to determine the adjusted effects of each variable and whether the impact of a specific variable remains after controlling for other factors.

### Qualitative data analysis

All qualitative interviews will be recorded, transcribed verbatim, and subsequently imported into ATLAS.ti, a qualitative software for coding and analysis [47]. A quality assurance protocol for qualitative analysis will be built into management and analysis, 25% of transcripts will be checked to verify accuracy of transcription, and 20% of transcripts will be double coded by Dr. Oluwoye and a trained research coordinator to ensure inter-coder reliability of ≥ 90%. We will use a direct content analysis to inform our analysis of interviews. A direct content analysis will include systematically assigning predetermined codes throughout the text [48]. Additional codes will be developed independently through an inductive process to identify themes that emerge from the data. To ensure consistency, a final codebook will be developed after several meetings prior to the final coding of transcripts. Resulting categories and themes will be summarized and used to inform essential elements needed for the interactive dashboard.

### Dissemination interactive dashboard development

Quantitative and qualitative data collected will be used to develop the web mapping visualization dashboard. Data regarding geographic accessibility to CSC programs, ADI, urban-rural status, ethnic density, and residential segregation will be aggregated at the census block group level and will be visualized using a web mapping dashboard interface. CSC programs throughout the U.S. will also be mapped. The dashboard will be created using d3 and OpenEpi JavaScript libraries, which are capable of creating advanced data-driven visualizations while incorporating statistical analysis functionalities. Aggregated data at the state, county, and block group level will enable users to zoom into different areas across the U.S. while simultaneously facilitating data analysis. Through display of univariate and bivariate statistics the dashboard will provide flexibility in exploring the data and a platform that will stimulate scientific insight. This visualization also combines Scalable Vector Graphics based web mapping (an XML-based vector image format with support for interactivity and animation) and highly compressed spatial data using Topojson technologies, creating benefits like quick loading times and a highly responsive interactive experience. Spatial and non-spatial data will be stored separately, housed on servers hosted by Washington State University, which will allow quicker data updates which will be maintained beyond the study period. This easy-to-use platform and its advanced analytical features will facilitate comparison of CSC program characteristics in different regions, generate new research questions, and validate or refute existing hypotheses.

## Discussion

By completing the proposed aims, this study will contribute to an increasing body of literature that explores the relationship between environmental and neighborhood-level determinants and the dissemination and implementation of CSC. Findings have the potential to inform policy surrounding future and existing CSC programs. Specifically, focusing on the funding and placement of CSC to improve the reach of programs to underserved communities. Additionally, the results from this study could be used to identify strategic points of intervention to improve access to and engagement in mental health services. Such information will facilitate strategic partnerships between state and local agencies with agencies or organizations in underserved areas. It will also help organizational leaders and providers understand barriers to CSC programs that have yet to be examined.

## Data Availability

The Washington State University and the study investigators are committed to the open and timely dissemination of research findings. The investigative team will ensure that the findings will be disseminated to CSC program directors, the National Association of State Mental Health Program Directors, and the NIMH-funded Early Psychosis Intervention Network (EPINET) National Data Coordinating Center for First Episode Psychosis at Westat.

## Abbreviations

CSC: Coordinated specialty care
FEP: First episode psychosis
GIS: Geographical information systems
NIMH: National Institute of Mental Health
NIMHD: National Institute of Minority Health and Health Disparities
RAISE-ETP: Recovery After an Initial Schizophrenia Episode-Early Treatment Program

## References

[1] Kirkbride JB, Fearon P, Morgan C, Dazzan P, Morgan K, Tarrant J (2006). Heterogeneity in incidence rates of schizophrenia and other psychotic syndromes: findings from the 3-center AeSOP study. Arch Gen Psychiatry..

[2] Kirkbride JB, Errazuriz A, Croudace TJ, Morgan C, Jackson D, Boydell J (2012). Incidence of schizophrenia and other psychoses in England, 1950–2009: a systematic review and meta-analyses. PloS One.

[3] Simon GE, Coleman KJ, Yarborough BJH, Operskalski B, Stewart C, Hunkeler EM (2017). First presentation with psychotic symptoms in a population-based sample. Psychiatr Serv.

[4] Clarke G, Yarborough BJ (2013). Evaluating the promise of health IT to enhance/expand the reach of mental health services. Gen Hosp Psychiatry.

[5] Glasgow RE, Vogt TM, Boles SM (1999). Evaluating the public health impact of health promotion interventions: the RE-AIM framework. Am J Public Health.

[6] Glasgow RE, Battaglia C, McCreight M, Ayele RA, Rabin BA (2020). Making implementation science more rapid: use of the RE-AIM framework for mid-course adaptations across five health services research projects in the Veterans Health Administration. Front Public Health.

[7] Shelton RC, Chambers DA, Glasgow RE (2020). An extension of RE-AIM to enhance sustainability: addressing dynamic context and promoting health equity over time. Front Public Health.

[8] Oluwoye O, Nagendra A, Kriegel LS, Anglin DM, Santos MM, López SR (2023). Reorienting the focus from an individual to a community-level lens to improve the pathways through care for early psychosis in the United States. SSM - Mental Health.

[9] Alegría M, Green JG, McLaughlin KA, Loder S. Disparities in child and adolescent mental health and mental health services in the US [Internet]. William T. Grant Foundation; 2015. Available from: https://scholar.google.com/scholar_lookup?hl=en&publication_year=2015&pages=%00empty%00&author=M.+Alegr%C3%ADa&author=J.+G.+Green&author=K.+A.+McLaughlin&author=S+Loder&isbn=%00null%00&title=Disparities+in+child+and+adolescent+mental+health+and+mental+health+services+in+the+US

[10] Cummings JR, Allen L, Clennon J, Ji X, Druss BG (2017). Geographic access to specialty mental health care across high- and low-income U.S. communities. JAMA Psychiatry.

[11] Oluwoye O, Amiri S, Kordas G, Fraser E, Stokes B, Daughtry R, et al. Geographic disparities in access to specialty care programs for early psychosis in Washington State. Adm Policy Ment Health. 2021;10.1007/s10488-021-01137-3 Cited 2021 Oct 18 10.1007/s10488-021-01137-3 PMC9119810 33877475

[12] Massey DS. America Becoming: Racial Trends and Their Consequences, Volume 1. Washington, D.C.: National Academies Press; 2001. p. 9599. Cited 2020 Dec 17. Available from: http://www.nap.edu/catalog/9599

[13] Bolin JN, Bellamy GR, Ferdinand AO, Vuong AM, Kash BA, Schulze A (2015). Rural Healthy People 2020: New Decade Same Challenges. J Rural Health.

[14] March D, Hatch SL, Morgan C, Kirkbride JB, Bresnahan M, Fearon P (2008). Psychosis and Place. Epidemiol Rev.

[15] Oluwoye O, Davis B, Kuhney FS, Anglin DM (2021). Systematic review of pathways to care in the U.S. for Black individuals with early psychosis. NPJ Schizophr.

[16] Dixon LB, Goldman HH, Srihari VH, Kane JM (2018). Transforming the treatment of schizophrenia in the United States: he RAISE initiative. Annu Rev Clin Psychol.

[17] Correll CU, Galling B, Pawar A, Krivko A, Bonetto C, Ruggeri M (2018). Comparison of early intervention services vs treatment as usual for early-phase psychosis: a systematic review, meta-analysis, and meta-regression. JAMA Psychiatry.

[18] Everett A, Heinssen R. Agency priority goal action plan: serious mental illness. Department of Health and Human Services; 2019 Cited 2020 Nov 18. Available from: https://www.performance.gov/health_and_human_services/2019_dec_HHS_Serious_Mental_Illness.pdf

[19] Rosenblatt, A. SAMHSA-funded programs for first episode psychosis across the United States. Bethesda; 2018.

[20] Kane JM, Schooler NR, Marcy P, Correll CU, Brunette MF, Mueser KT (2015). The RAISE early treatment program for first-episode psychosis: background, rationale, and study design. J Clin Psychiatry.

[21] Oluwoye O, Stiles B, Monroe-DeVita M, Chwastiak L, McClellan JM, Dyck D (2018). Racial-ethnic disparities in first-episode psychosis treatment outcomes from the RAISE-ETP study. Psychiatr Serv.

[22] Tabak RG, Khoong EC, Chambers DA, Brownson RC (2012). Bridging research and practice: models for dissemination and implementation research. Am J Prev Med.

[23] Purtle J, Nelson KL, Bruns EJ, Hoagwood KE (2020). Dissemination strategies to accelerate the policy impact of children’s mental health services research. PS..

[24] Purtle J, Lê-Scherban F, Nelson KL, Shattuck PT, Proctor EK, Brownson RC (2020). State mental health agency officials’ preferences for and sources of behavioral health research. Psychol Serv.

[25] Hoagwood KE, Purtle J, Spandorfer J, Peth-Pierce R, Horwitz SM (2020). Aligning dissemination and implementation science with health policies to improve children’s mental health. Am Psychol.

[26] Eccles MP, Mittman BS (2006). Welcome to Implementation Science. Implement Sci.

[27] Nilsen P, Bernhardsson S (2019). Context matters in implementation science: a scoping review of determinant frameworks that describe contextual determinants for implementation outcomes. BMC Health Serv Res.

[28] Watson DP, Adams EL, Shue S, Coates H, McGuire A, Chesher J (2018). Defining the external implementation context: an integrative systematic literature review. BMC Health Serv Res.

[29] Michie S, Johnston M, Abraham C, Lawton R, Parker D, Walker A (2005). Making psychological theory useful for implementing evidence based practice: a consensus approach. Qual Saf Health Care.

[30] Cane J, O’Connor D, Michie S (2012). Validation of the theoretical domains framework for use in behaviour change and implementation research. Implement Sci.

[31] Damschroder LJ, Aron DC, Keith RE, Kirsh SR, Alexander JA, Lowery JC (2009). Fostering implementation of health services research findings into practice: a consolidated framework for advancing implementation science. Implement Sci.

[32] Beidas RS, Edmunds J, Ditty M, Watkins J, Walsh L, Marcus S (2014). Are inner context factors related to implementation outcomes in cognitive-behavioral therapy for youth anxiety?. Adm Policy Ment Health..

[33] Williams NJ, Frank HE, Frederick L, Beidas RS, Mandell DS, Aarons GA (2019). Organizational culture and climate profiles: relationships with fidelity to three evidence-based practices for autism in elementary schools. Implement Sci.

[34] Aarons GA, Glisson C, Green PD, Hoagwood K, Kelleher KJ, Landsverk JA (2012). The organizational social context of mental health services and clinician attitudes toward evidence-based practice: a United States national study. Implement Sci.

[35] Escoffery C, Sekar S, Allen CG, Madrigal L, Haardoerfer R, Mertens A (2023). A scoping review of outer context constructs in dissemination and implementation science theories, models, and frameworks. Transl Behav Med.

[36] Alvidrez J, Castille D, Laude-Sharp M, Rosario A, Tabor D (2019). The National Institute on Minority Health and Health Disparities Research Framework. Am J Public Health.

[37] US Government Publishing Office. Public Law 106–525. Cited 2020 Nov 22. Available from: https://www.govinfo.gov/content/pkg/PLAW-106publ525/pdf/PLAW-106publ525.pdf

[38] Oluwoye O, Kriegel L, Alcover KC, Compton MT, Cabassa LJ, McDonell MG (2020). The impact of early family contact on quality of life among non-Hispanic Blacks and Whites in the RAISE-ETP trial. Schizophrenia Res.

[39] Anglin DM, Lighty Q, Greenspoon M, Ellman LM (2014). Racial discrimination is associated with distressing subthreshold positive psychotic symptoms among US urban ethnic minority young adults. Soc Psychiatry Psychiatr Epidemiol.

[40] Cummings JR, Case BG, Ji X, Marcus SC (2016). Availability of youth services in U.S. mental health treatment facilities. Adm Policy Ment Health.

[41] Substance Abuse and Mental Health Administration. National Mental Health Services Survey (N-MHSS): 2019, Data On Mental Health Treatment Facilities | CBHSQ Data. Available from: https://www.samhsa.gov/data/report/national-mental-health-services-survey-n-mhss-2019-data-mental-health-treatment-facilities

[42] Palinkas LA, Horwitz SM, Green CA, Wisdom JP, Duan N, Hoagwood K (2015). Purposeful sampling for qualitative data collection and analysis in mixed method implementation research. Adm Policy Ment Health.

[43] McGrail MR (2012). Spatial accessibility of primary health care utilising the two step floating catchment area method: an assessment of recent improvements. Int J Health Geographics.

[44] Guagliardo MF (2004). Spatial accessibility of primary care: concepts, methods and challenges. Int J Health Geogr.

[45] Schuurman N, Bérubé M, Crooks VA (2010). Measuring potential spatial access to primary health care physicians using a modified gravity model. Can Geographer / Le Géographe canadien.

[46] McGrail MR, Humphreys JS, Ward B (2015). Accessing doctors at times of need–measuring the distance tolerance of rural residents for health-related travel. BMC Health Serv Res.

[47] Muhr T. ATLAS. ti 6.0 [version 6]. Berlin, Germany: Scientific Software Development GmbH; 2004.

[48] Hsieh H-F, Shannon SE (2005). Three approaches to qualitative content analysis. Qual Health Res.

